# Low power flexible monolayer MoS_2_ integrated circuits

**DOI:** 10.1038/s41467-023-39390-9

**Published:** 2023-06-19

**Authors:** Jian Tang, Qinqin Wang, Jinpeng Tian, Xiaomei Li, Na Li, Yalin Peng, Xiuzhen Li, Yanchong Zhao, Congli He, Shuyu Wu, Jiawei Li, Yutuo Guo, Biying Huang, Yanbang Chu, Yiru Ji, Dashan Shang, Luojun Du, Rong Yang, Wei Yang, Xuedong Bai, Dongxia Shi, Guangyu Zhang

**Affiliations:** 1 Beijing National Laboratory for Condensed Matter Physics and Institute of Physics, Chinese Academy of Sciences, Beijing, 100190 China; 2 School of Physical Sciences, University of Chinese Academy of Sciences, Beijing, 100190 China; 3 Shanghai Key Laboratory of Multidimensional Information Processing, East China Normal University, Shanghai, China; 4 Songshan Lake Materials Laboratory, Dongguan, 523808 China; 5 Institute of Advanced Materials, Beijing Normal University, Beijing, 100875 China; 6 Institute of Microelectronics, Chinese Academy of Sciences, Beijing, 100029 China

**Keywords:** Electronic devices, Electronic devices

## Abstract

Monolayer molybdenum disulfide (ML-MoS_2_) is an emergent two-dimensional (2D) semiconductor holding potential for flexible integrated circuits (ICs). The most important demands for the application of such ML-MoS_2_ ICs are low power consumption and high performance. However, these are currently challenging to satisfy due to limitations in the material quality and device fabrication technology. In this work, we develop an ultra-thin high-κ dielectric/metal gate fabrication technique for the realization of thin film transistors based on high-quality wafer scale ML-MoS_2_ on both rigid and flexible substrates. The rigid devices can be operated in the deep-subthreshold regime with low power consumption and show negligible hysteresis, sharp subthreshold slope, high current density, and ultra-low leakage currents. Moreover, we realize fully functional large-scale flexible ICs operating at voltages below 1 V. Our process could represent a key step towards using energy-efficient flexible ML-MoS_2_ ICs in portable, wearable, and implantable electronics.

## Abstract (web summary)

The application of 2D MoS_2_ flexible integrated circuits (ICs) is currently limited by the material quality over large areas and the device fabrication technology. Here the authors report a gate-first fabrication technique to realize wafer-scale monolayer MoS_2_ ICs on rigid and flexible substrates with high performance and low power consumption.

## Introduction

Flexible electronics plays an integral role in a large spectrum of fields including information technology, energy generation and storage, bio-sensing, and diagnosis^1–5^. Among them, flexible integrated circuits (ICs) dealing with information processing are favorable in portable, wearable, and implantable electronics with technological demands towards flexibility and robustness of large-area devices^6–8^. Conventional flexible ICs are usually fabricated from organic semiconductors^6, 8^, silicon of either amorphous or polycrystalline forms^9,10^, oxide semiconductors^11,12^, and carbon nanotubes (CNTs)^13–17^ via the thin-film-transistor (TFT) technology. Recently, the 2D semiconductor of monolayer MoS_2_ (ML-MoS_2_) emerged as an advanced channel material in large-area flexible TFTs^18–26^. In principle, such TFTs have great potential in both high performance and low-power applications, if considering the following merits. First, ML-MoS_2_ is atomically thin (only ~0.7 nm) and smooth yet mechanically strong (in-plane) and flexible (out-of-plane)^1,27^. Such a thin channel also offers benefits in ultra-scale devices where short channel effects would be the main concern^28–30^. Second, its 2H phase has a moderate band gap of ~2 eV, between that of silicon (~1.1 eV) and indium gallium zinc oxide (~3.5 eV), hence can work with both low off-state and high on-state currents^31,32^. Third, it has high electrical quality and is available at wafer scale^22,33–36^.

It has been shown that, at room temperature, rigid ML-MoS_2_ TFTs can feature high electron mobility (*μ*) of >100 cm^2^·V^−1^·s^−1^, high on/off ratio of >10^8^, low subthreshold swing (SS) approaching the thermionic limit of 60 mV·dec^−1^, and high on-current of ~1.1 mA·μm^−1^ at a supply voltage of 1.5 V^37–39^. Hence, one might ask if such TFT technologies developed on rigid substrates could be transferred onto flexible substrates and be superior to existing flexible TFT technologies. Up to now, large-scale flexible ML-MoS_2_ ICs with various fully functional units such as logic gates, inverters, and ring oscillators (ROs) have been demonstrated vividly^23–25^. However, limited by materials and fabrications, such large-area flexible ICs suffer from either low performances or high-power consumptions, and it is challenging to realize both in a synergic manner.

In this work, we demonstrate the realization of low-power flexible ICs based on ML-MoS_2_. The technological advances of this work lay on both the newly developed high-quality ML-MoS_2_ wafers and the ultra-thin high-κ dielectric/metal-gate technology. High-quality 4-inch wafer-scale ML-MoS_2_ films are epitaxially grown on sapphire via an oxygen-assisted chemical vapor deposition (CVD) approach we developed recently^34^. As-grown films are strictly monolayer, uniform across entire substrates, and have large domain sizes over 200 μm. Such high-quality materials offer a desirable choice for realizing large-scale flexible ICs. Here, the flexible ICs are fabricated via a gate-first technology in which ultra-thin high-κ dielectrics are deposited on gate electrodes to achieve a record-low equivalent oxide thickness (EOT). Due to the enhanced gating efficiency, our ML-MoS_2_ flexible TFTs can work under operation voltages below 1 V.

## Results

### Gate-first technology for ultra-thin high-κ dielectric deposition

**Fig. 1:**
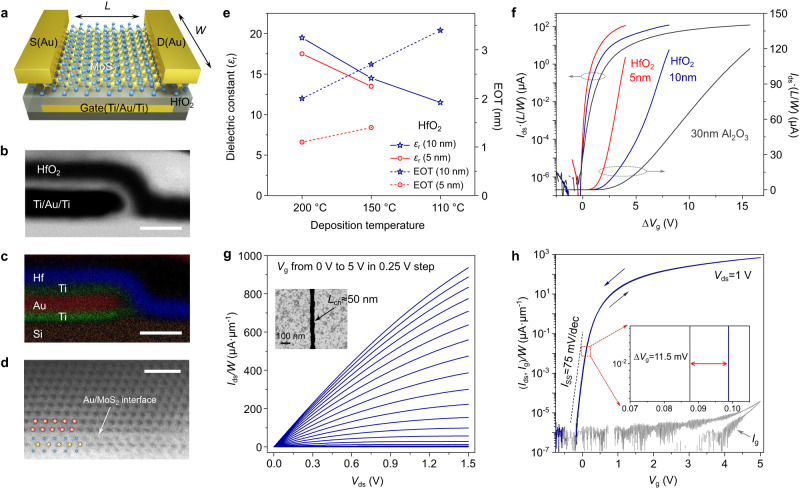
Characterizations of monolayer MoS_2_ thin-film-transistor (TFTs) on SiO_2_ substrates. **a** Schematic illustration of a buried-gate MoS_2_ TFT. **b**, **c** Cross-sectional scanning transmission electron microscope (STEM) image (**b**) of 5-nm HfO_2_ on the buried Ti-Au-Ti (1-5-1 nm) gate electrode, and corresponding energy disperse spectroscopy (EDS) elemental mapping image (**c**) clearly shows the distribution of Au, Ti, Hf and Si elements. Scale bars, 10 nm. **d** Atomic-resolution cross-sectional STEM image of Au-MoS_2_ contact interface. Red, yellow, and blue spheres are Au, Mo, and S atoms, respectively. Scale bar, 1 nm. **e** The dielectric constant and equivalent oxide thickness (EOT) of HfO_2_ layers deposited at different temperatures. **f** Normalized transfer curves of MoS_2_ TFTs (*L* = 5 μm, *W* = 40 μm) at *V*_ds_ = 1 V with 5 nm, 10 nm HfO_2_ and 30 nm Al_2_O_3_ as the dielectric layer, individually. **g**, **h** Typical electrical output and transfer curves of MoS_2_ TFTs with *L*_ch_ ≈ 50 nm and 5-nm HfO_2_ as the dielectric layer. Inset to (**g**) is the scanning electron microscope (SEM) image of the *L*_ch_ ≈ 50 nm device. The *I*_ds_/*W*-*V*_g_ curve features with the sharp subthreshold swing (*I*_SS_ = 75 mV·dec^−1^, refer to the black dash line) and small hysteresis (∆*V*_g_ = 11.5 mV, inset to **h**). The leakage current (*I*_g_) is represented in gray color in (**h**).

In a power-efficient system, transistors and circuits should ideally have a low-driving voltage which can be created by using high-capacitance dielectric layers for efficient electrostatic gating. We thus employed ultra-thin high-κ dielectrics of HfO_2_, a technique widely used in the present silicon-based high-performance and low-power electronics. Since ultra-thin high-κ dielectrics are very difficult to deposit on MoS_2_ due to the lack of surface dangling bonds^40,41^, we hence developed the high-κ dielectric/metal-gate technology, i.e. a gate-first technology, for ML-MoS_2_ TFTs. Figure 1a illustrates the device geometry with buried Ti-Au-Ti as the local back-gate electrode, ultra-thin HfO_2_ as the dielectric layer, ML-MoS_2_ as the channel, and Au as the source/drain electrodes.

During the device gate-first fabrication process, buried-gate electrodes of Ti-Au-Ti (1-5-1 nm) were first deposited on a substrate (either rigid or flexible) by standard lithography and electron beam evaporation processes. Note that the top 1 nm-Ti layer after oxidation in O_2_ plasma serves as the seeding layer for following atomic layer deposition (ALD) of the high-κ dielectrics; while the bottom 1 nm-Ti layer acts as the adhesive layer between Au and substrate. Other than the deposition of the seeding layer for the subsequent HfO_2_ deposition on 2D materials like graphene^42^, our approach employs the seeding layer on metal gates which are more general and compatible with the semiconductor fabrication process. Before and after metal gate depositions, we performed oxygen plasma cleaning to remove the photoresist residues on side walls and top surface of patterns/metal-gates introduced from the lithography process, as illustrated in Supplementary Fig. 1. With the help of this cleaning process, we can produce flat metal gates with clean surfaces and sharp boundaries. After ALD, the deposited HfO_2_ layers on metal gates are very uniform, as characterized by the atomic force microscope (AFM). The measured surface roughness of 5-nm HfO_2_ deposited on Ti/Au/Ti metal gates is typically <0.5 nm. After high-κ dielectric deposition, a 4''-wafer-sized ML-MoS_2_ film (See Supplementary Fig. 2 and Table 1 for film quality characterizations and comparisons) was transferred onto the overall structure by a wet chemical etching, and the transferred ML-MoS_2_ films are almost intact and flat on target substrates process (see Methods and Supplementary Fig. 3). Finally, multistep lithography, reactive ion etching (RIE), and electron beam evaporation were performed to define the channel and source/drain (S/D) contact regions.

For a typical TFT on the SiO_2_ substrate, we characterized its interfaces between different layers by cross-sectional scanning transmission electron microscope (STEM). Figure 1b, c shows a typical STEM image and corresponding Au, Ti, Hf, Si elemental mapping from energy disperse spectroscopy (EDS) at the HfO_2_/metal-gate interface. We can see a uniform and conformal coating of HfO_2_ with a thickness of 5 nm on the metal gate. Figure 1d shows an atomic-resolution STEM image of the Au-MoS_2_ interface. The sharp interface between periodically arranged Au atoms and three-atom-thick MoS_2_ surface without any defects or cracks suggests the high quality of contacts.

To evaluate the dielectric properties of HfO_2_ layers deposited on metal gates by ALD, we performed capacitance measurements (Supplementary Fig. 4). In Fig. 1e, we plot the dielectric constants (*ε*_r_) of 5-nm and 10-nm thick HfO_2_ layers deposited at 110, 150, and 200 °C. Obviously, higher deposition temperature and thicker thickness are beneficial to achieving a better dielectric property. Hence, the ALD temperature of 200 °C was applied for rigid devices; whereas it is 150 or 110 °C for flexible devices to reduce the temperature-induced substrate deformation. In our batch fabrication of devices, the minimum HfO_2_ thickness (*t*_HfO2_) is 5 nm on rigid substrates, e.g., SiO_2_, and 10 nm on flexible substrates, e.g., polyethylene terephthalate (PET, see Supplementary Fig. 5 for roughness characterizations), for a reliable device yield. Note that the 5-nm HfO_2_ layer has an effective oxide thickness (EOT) of only 1.1 nm. Such low EOT is beneficial to high device transconductance at low supply voltages and efficient gating of MoS_2_ channels.

### ML-MoS_2_ TFTs on rigid substrates

Firstly, let’s evaluate ML-MoS_2_ TFTs on SiO_2_ substrates with normalized channel lengths (*L*_ch_) and widths (*W*). Figure 1f shows the normalized transfer curves (*I*_ds_·*L*/*W*-∆*V*_g_) of devices with HfO_2_ layer thickness of 5 and 10 nm. As a comparison, 30 nm thick Al_2_O_3_ devices are also included. It can be clearly seen that with decreased *t*_HfO2_ and increased *ε*_r_, the gate voltages (*V*_g_) can be reduced from 15 V to 3 V and the SS can be reduced from 250 mV·dec^−1^ to 75 mV·dec^−1^, while preserving the on-state current (*I*_on_) densities and on/off ratios.

Figure 1g, h demonstrate typical output (*I*_ds_/*W*-*V*_ds_) and transfer curves (*I*_ds_/*W*-*V*_g_) of a short channel device with *t*_HfO2_ = 5 nm and *L*_ch_ ≈ 50 nm. This device features ultra-high on/off ratio of ~10^9^, sharp subthreshold slope (SS) of *I*_SS_ = 75 mV·dec^−1^ over 5 orders of magnitude, and negligible hysteresis (∆*V*_g_ ~ 11.5 mV, inset to Fig. 1h) subjected to the high quality of MoS_2_/HfO_2_ interface. The ultra-high on/off ratio could guarantee an ultra-low static power dissipation. When devices work at a stand-by mode (off state), the leakage current (*I*_g_/*W*) can reach below 1 pA·μm^−1^ (Fig. 1h) for a general *W*/*L*_ch_ = 5 μm/50 nm device. Due to the high gating efficiency, the maximum field could approach ~1 V·nm^−1^ and the carrier density can approach *n*_*i*_ = 5.15 × 10^13 ^cm^−2^ at *V*_g_ = 5 V. Note that the effective carrier densities in MoS_2_ channel would be overestimated based on the metal-insulator-metal capacitance (MIMCAP) results, thus we carried out metal-oxide-semiconductor capacitance (MOSCAP) measurements based on the MoS_2_ FET (Supplementary Fig. 6). Such high carrier densities in ML-MoS_2_ channels are among the highest in previous reports. As a result, the maximum on-state current densities (*I*_on_/*W*) could reach 936 μA·μm^−1^ at *V*_ds_ = 1.5 V with *L*_ch_ = 50 nm in Fig. 1g, which is comparable to the highest value (1135 μA·μm^−1^ at *V*_ds_ = 1.5 V) previously achieved in Bi-contacted ML-MoS_2_ TFTs with *L*_ch_ = 35 nm^39^. More data on MoS_2_ TFTs with *L*_ch_ of 150 nm and 300 nm appears in Supplementary Fig. 7. Note that even Au-contacts show slightly higher contact resistances than Bi-contacts, our devices still show linear output characteristics at small bias and saturated output behaviors at high bias voltages, which is important for the output current capacity of short channel devices. Besides, *I*_on_/*W* is ~720 μA·μm^−1^ at *V*_ds_ = 1 V, meeting the low-power application metrics of the International Roadmap for Devices and Systems (IRDS, 2024)^43^.

### ML-MoS_2_ TFTs on flexible substrates

**Fig. 2:**
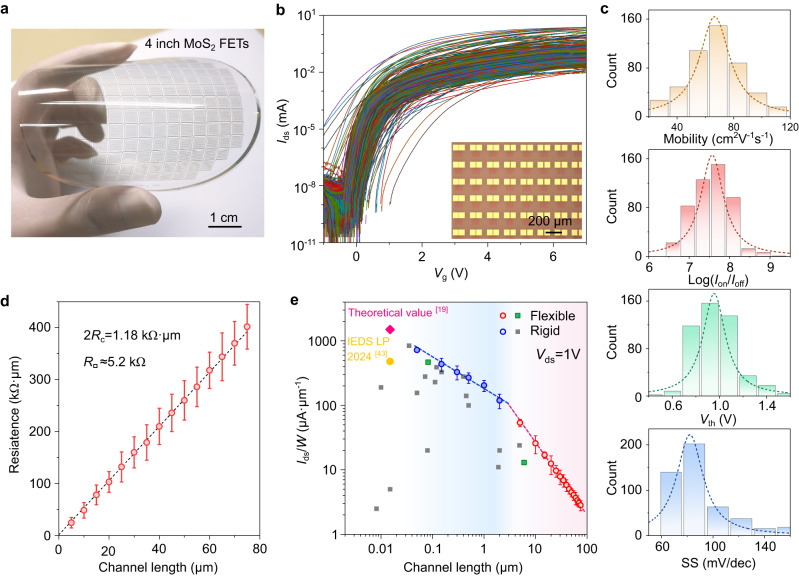
Flexible ML-MoS_2_ TFTs and electrical properties. **a** Photograph of an as-fabricated 4-inch MoS_2_ TFTs on the polyethylene terephthalate (PET) substrate. Scale bar, 1 cm. **b** Transfer curves of 500 randomly picked TFTs with the 10 nm HfO_2_ dielectrics. Bias voltage (*V*_ds_) is 1 V, and the device yield is 96.2%. Inset to (**b**) shows the enlarged image of the device array. Scale bar, 200 μm. **c** Histogram and Lorentz distribution fits of the device mobility, on/off ratio, threshold voltage and subthreshold swing extracted from the transfer curves in (**b**). **d** Contact and sheet resistance measurements from transfer length method (TLM) at *n*_i_ ≈ 2.8 × 10^13 ^cm^−2^. **e** Statistics and comparisons of the on-state current density of the flexible and rigid TFTs at *V*_ds_ = 1 V versus channel length. This work is highlighted in red and blue circles, while the reference data points are drawn in gray and green squares (please refer to Supplementary Tables 2, 3 for details). Error bars in (**d**) and (**e**) are taken from 10 transistors with the same channel length.

Next, we implement the technique of ultra-thin high-κ dielectric/metal gate deposition on flexible PET substrate. Figure 2a shows 4” wafer-scale ML-MoS_2_ TFTs on PET substrate. Now let’s evaluate devices on flexible substrates based on 10 nm HfO_2_. These long-channel TFTs have a high device yield (>96%) and nice spatial uniformity. In Fig. 2b we show transfer curves of 500 randomly picked TFTs with *L*_ch_ varying from 5 to 75 μm. Statistics on the device’s *μ*_FE_, on/off ratio, threshold voltage (*V*_th_), and SS are shown in Fig. 2c. According to Lorentz distribution fittings, *μ*_FE_ averages at ~70 cm^2^·V^−1^·s^−1^ (maximizes at >110 cm^2^·V^−1^·s^−1^); on/off ratio averages at 5 × 10^7^ (maximizes at ~1 × 10^9^); *V*_th_ is centered at 0.96 ± 0.4 V; and SS averages at 83 mV·dec^−1^. All these performances are comparable to those previously achieved in rigid ML-MoS_2_ TFTs (Supplementary Table 2) and greatly improved over the previous state-of-the-art flexible TFTs (Supplementary Table 3). It is worth noting here that both the positive *V*_th_ and small SS are critical for the low power consumption in our devices, as will be shown later. Note that we also realize the fabrications of flexible ML-MoS_2_ FETs based on 5 nm HfO_2_ as measured in Supplementary Figs. 8, 9. Considering the low device yield (~60%) and higher leakage current level of the 5 nm HfO_2_, we mainly adopt 10 nm HfO_2_ as dielectric layer for integrated logic gates in following experiments.

According to previous studies^44^, the *V*_th_ variability comes mainly from sources including material uniformity (defects, grain boundaries, and layer thicknesses), oxide roughness, surface (MoS_2_ surface), and interface (contact interface, oxide-MoS_2_ interface) cleanness. In this study, MoS_2_ is strictly monolayer without additional layers and of high quality. High defects (mainly S-vacancies) density could cause severe n-doping in the devices, shifting *V*_th_ to very negative values. In present devices, the positive *V*_th_ reflects the low density of vacancy defects in the MoS_2_ channel region. The domain sizes in our samples are over 200 µm, much larger than the channel sizes, suggesting that grain boundaries are also not likely the source of *V*_th_ variations. The thin HfO_2_ layers deposited on metal gates are smooth with a surface roughness of ~0.5 nm, measured by AFM. We thus conclude that the main source of *V*_th_ variability, although it is small, comes mainly from the surface and interface cleanness.

The high device performance is attributed to several aspects. First, our ML-MoS_2_ films are of high quality. The extracted sheet resistance *R*_□_ is ~5.2 kΩ at *n*_i_ ≈ 2.8 × 10^13 ^cm^−2^ fitted by the transfer length method (TLM), as shown in Fig. 2d. Second, the employment of HfO_2_ dielectrics allows us to tune carrier densities in MoS_2_ over than 5 × 10^13 ^cm^−2^. Third, the contact resistance *R*_c_ between Au and MoS_2_ is as low as ~0.59 kΩ·μm (see Fig. 2d and Supplementary Fig. 10 for the *R*_c_ extraction based on long channel and short channel devices). Such low *R*_c_ is facilitated by the ultra-slow Au-deposition rates (see Methods) and the bottom gate structure in which the contact region of ML-MoS_2_ can be doped to be metallic properties. Based on our data from both rigid and flexible TFTs, here we highlight the on-state current densities (*I*_ds_/*W*) as a function of *L*_ch_ in Fig. 2e. Clearly, our data points lay at the upper envelope boundary if compared with literature works from monolayer flexible or rigid MoS_2_ TFTs (all reference data points acquired from Supplementary Tables 2, 3).

### Low-power and high-performance flexible ICs

**Fig. 3:**
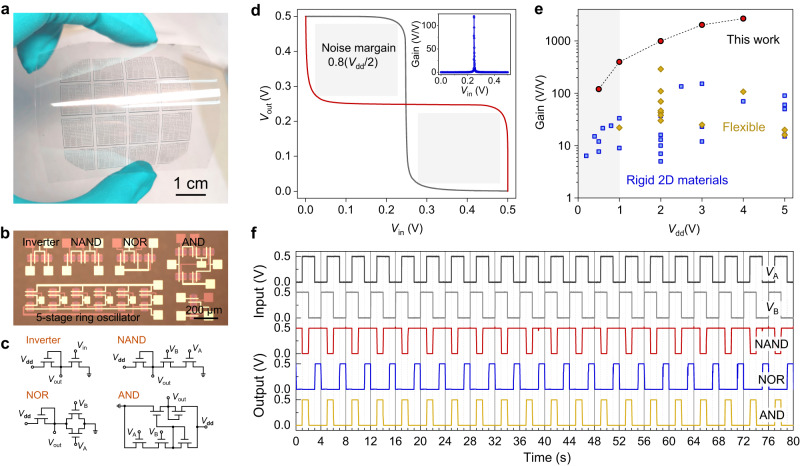
Flexible integrated circuits at low driving voltages. **a** Photograph of 4 × 4 cm^2^ integrated monolayer MoS_2_ TFTs on the flexible PET substrate. Scale bar, 1 cm. **b**, **c** Optical image (**b**) and schematic circuits (**c**) of logic inverters, NAND, NOR, AND gates and 5-stage ring oscillators. Scale bar, 200 μm. *V*_dd_, power supply voltage; *V*_in_, *V*_A_, or *V*_B_, input voltages; *V*_out_, output voltage. **d** Voltage transfer curves (VTCs) of a logic inverter with noise margin of 0.8(*V*_dd_ /2) at *V*_dd_ = 0.5 V. Inset shows corresponding voltage gain of 120. **e** Comparison of the inverter voltage gain as a function of *V*_dd_ among flexible inverters or rigid inverters based on 2D materials. The reference data points are listed in Supplementary Table 4. **f** Output characteristics of logic NAND, NOR, AND gates as a function of input voltage pulses with *V*_dd_ = 0.5 V.

We further fabricated large-area flexible ICs based on ML-MoS_2_. Figure 3a shows a photograph of 4 × 4 cm^2^ electronic circuits fabricated on the PET substrate. As logic gates and inverters are essential building blocks of ICs, we included logic inverters, NAND, NOR, and AND gates in this batch (Fig. 3b, c).

The output characteristic of ML-MoS_2_ inverter shows abrupt switching behavior with power supply voltages *V*_dd_ = 0.5 V (Fig. 3d). The trip point of the inverter is when output voltage (*V*_out_) equals input voltage (*V*_in_) and of particular importance for low-power electronics as it determines the driving voltage of integrated logic circuits. The typical inverter also exhibits full swing output behavior with noise margin (NM) of 0.8×(*V*_dd_ /2) and voltage gain of 120. Note that the highest voltage gain is 192 at *V*_dd_ = 0.5 V and 397 at *V*_dd_ = 1 V, and it can approach 1000 (2670) at *V*_dd_ = 2 V (4 V), the highest ever achieved in MoS_2_ inverters (see Supplementary Fig. 11). Note that the rail-to-rail operations could be realized by further optimizing the circuit design of the FET units^45^, combing p-type FETs as building blocks^46^ or introducing doping techniques^47^. As can be seen in Fig. 3e, our MoS_2_ inverters have ultra-high gains and reliable outputs within the sub-1V supply voltage zone, as compared with other flexible inverters or rigid inverters based on 2D materials (the reference data points acquired from Supplementary Table 4). The ultra-high voltage gain is enabled by the strong gate controllability, while the trip point is mainly determined by the V_th_ distribution in our MoS_2_ TFTs. We also analyzed the individual TFT unit within an inverter (Supplementary Fig. 12) with intrinsic gains^48^ (defined as *A*_i_ = *g*_m_/*g*_d_) from 10^3^ to 10^4^, where *g*_m_ and *g*_d_ are the transconductance and output conductance of the TFT. Through monitoring the channel current of an inverter, the MoS_2_ TFT unit works mainly at the deep-subthreshold regime with ultra-low current densities. The calculated maximum output power of an inverter is 10.3 pW·μm^−1^, at the same level as an oxide semiconductor inverter^32^; and the quiescent power is below 0.1 pW·μm^−1^ at *V*_dd_ = 1 V (see Supplementary Table 5 for power consumption comparisons).

Typical output characteristics of ML-MoS_2_ logic NAND, NOR, AND gates are also shown in Fig. 3f. 0.5 V pulses of *V*_A_ and *V*_B_ with 1 s delay are used as input signals, where 0.5 V for the logic ‘1’ and 0 V for the logic ‘0’. All logic gates have correct Boolean output functionalities with a low supply voltage *V*_dd_ = 0.5 V.

**Fig. 4:**
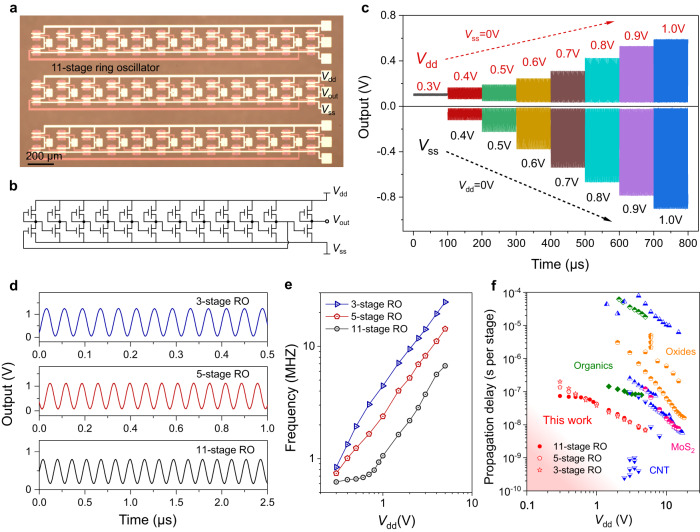
Flexible ML-MoS_2_ ring oscillators (ROs) operated at sub-1 V supply voltages. **a**, **b** Optical image and schematic circuit diagram of 11-stage ROs. Scale bar, 200 μm. *V*_dd_, or *V*_ss_, power supply voltages applied on drain or source electrodes. **c** Output signals of an 11-stage RO operated with sub-1 V supply voltages from 0.3 V to 1 V. **d** Output signals of 3-stage, 5-stage and 11-stage ROs operated at *V*_dd_ = 5 V. The output oscillation frequency increases by decreasing the stage number. **e** Summarized output frequencies of ROs as a function of *V*_dd_ from 0.3 V to 5 V. The output oscillation frequency increases with supply voltages. **f** Comparisons of propagation stage delay time as well as supply voltages with literature works. Detailed parameters are listed in Supplementary Table 6.

In addition to logic gates and inverters, flexible ML-MoS_2_ ROs with various number of stages were also fabricated to demonstrate the reliability for low voltage operations. Figure 4a shows the optical microscope image of 11-stage ROs which integrate cascading eleven inverters and an additional inverter as the output buffer for measurements. The corresponding schematic circuit with three terminal electrodes of *V*_dd_, *V*_ss_, and *V*_out_ is shown in Fig. 4b. Figure 4c shows the stable electrical output signals of an 11-stage RO at supply voltage *V*_dd_ from 0.3 V to 1 V (*V*_ss_ terminal is grounded). Such low-driving voltages are facilitated from the small trip point of logic inverters. In contrast, if negative voltages are applied on the *V*_ss_ terminal while the *V*_dd_ terminal is grounded, the RO could output negative signals starting from *V*_ss_ = −0.4 V, as the swing threshold voltages of logic inverters shift negatively with the decreasing the operation voltages of *V*_ss_ or *V*_dd_ terminal (Supplementary Fig. 11c, d).

Generally, the output amplitude increases with the supply voltages and the oscillation frequency (*f*) decreases with the number of stages. Figure 4d shows the maximum output oscillation signals of 3-, 5- and 11-stage ROs operated at *V*_dd_ = 5 V. The ROs have reliable outputs with supply voltages varying from 0.3 V to 5 V (Fig. 4e). The maximum *f* is 24.8, 14.29, and 6.7 MHZ for 3-, 5- and 11-stage ROs, respectively. The corresponding propagation stage delay time (*τ*_pd_ = 1/2*Nf*, where *N* is RO stage number) at *V*_dd_ = 5 V is 6.7, 7.0, and 6.8 ns for 3-, 5-, and 11-stage ROs, respectively. Compared with previous flexible ROs fabricated from other 2D materials, organic, oxides or CNTs, our ROs locate at the preferred conner with the low driving voltages (sub-1 V) and fast propagation stage delay time (Fig. 4f). Note that, in this work, the parasitic capacitance from the overlapping region between the contact electrodes/additional outer MoS_2_ region with the gate is detrimental to high-frequency operation. The typical channel length/width of TFT unit is 1 μm/3 μm for ROs, and more device details and parasitic capacitance optimization is demonstrated in Supplementary Fig. 13. A typical real-time outputs of oscillating RO is included in the Supplementary Movie to show the stable operation.

Finally, we performed bending tests of the flexible ML-MoS_2_ ICs. As an example, here we show results from flexible MoS_2_ TFTs and 5-stage RO in Supplementary Figs. 14–17. On/off ratios as well as field-effect mobilities of individual TFTs could be well-preserved under a minimum bending radius (*R* = 2.4 mm). Under such bending, either along the X- or Y- direction, the 5-stage RO exhibits stable outputs without obvious degradations. The excellent mechanical flexibility and endurance, reliable electrical outputs of ROs, and high device yield indicate the good device performances and uniformity of our devices fabricated on flexible substrates; and the lowest operation supply voltage indicates the potential for low-power electronic applications.

## Discussion

We developed an ultra-thin high-κ dielectric on metal gate technology for ML-MoS_2_ TFTs. Benefiting from the high-quality ML-MoS_2_ wafers as well as ohmic contacts, our ML-MoS_2_ TFTs could be tuned to high carrier density of 5.15 × 10^13 ^cm^−2^ and allow a high current capacity of 936 μA·μm^−1^ at *V*_ds_ = 1.5 V with sharp SS of 75 mV·dec^−1^, positive threshold voltages, negligible hysteresis, and ultra-low leakage currents. Large-area flexible TFTs and ICs show excellent spatial uniformity and a high device yield of >96% with fully functional inverters, logic gates, and ring oscillators working reliably under supply voltages below 1 V. The power consumption of an inverter can be as low as 10.3 pW·μm^−1^ at *V*_dd_ = 1 V. Besides, both the voltage gains of inverters and the propagation delay time of ROs we achieved are record-high values against previous flexible devices. Our results suggest that ML-MoS_2_ is a very competitive channel material in flexible ICs for both high performance and low power applications.

## Methods

### Growth of high-quality ML-MoS_2_ films

The growth was carried out in a CVD system using S (Alfa, 99.5%, 8 g) and MoO_3_ (Alfa, 99.9995%, 30 mg) powder as reaction sources. 4-inch c-plane sapphire wafers were used as substrates. In order to achieve large domain sizes, we intended to reduce the nucleation density during the growth process and elongate the growth time accordingly. The nucleation density of MoS_2_ was reduced by using higher oxygen flow rate for MoO_3_ source and reducing the Mo-source flux by lowering its evaporation temperature, increasing the distance between the Mo-source and sapphire substrate. During the growth, carrier gases of Ar (40 sccm) and Ar (240 sccm)/O_2_ (10 sccm) were fluxed for S power and MoO_3_ individually and the pressure in the chamber is ~1 Torr; temperatures were hold at 130 °C, 530 °C, and 930 °C for the S-, MoO_3_-source and substrate; and the growth process lasts usually about 50 min. Supplementary Fig. 2 demonstrates an optical image of high-quality 4-inch ML-MoS_2_ wafer with large domain sizes between 200 μm and 500 μm. These domains are well-stitched together to form a continuous film with 100% coverage.

### Deposition of High-κ dielectric layers

ALD of HfO_2_ on Ti/Au/Ti (1 nm/5 nm/1 nm) local metal gates were carried out by Savannah-100 system (Cambridge NanoTech. Inc.) with H_2_O and tetrakis dimethylamino hafnium (TDMAH) as precursors. Prior to the deposition process, we used O_2_ plasma to treat the Ti/Au/Ti electrode surface by reactive ion etching (RIE) to oxidize the surface Ti layer. The oxidized layer (TiO_2-*x*_) acts as a buffer layer for dense high-κ dielectrics deposition. During the deposition, 20 sccm high-purity nitrogen was flowed as the carrier and purge gas; the reactor pressure was ~3 Torr; and the TDMAH precursor was heated to 75 °C. The deposition temperature is 110 °C or 150 °C for flexible samples, and 200 °C for rigid samples. The pulse and reaction time was 0.015/0.15 and 60/60 s for TDMAH/H_2_O precursors with deposition rate around 1 Å per cycle.

### Device fabrications and measurements

TFTs and logic devices were fabricated via standard microfabrication processes such as e-beam lithography or UV lithography, oxygen RIE, e-beam evaporation and lifting-off. Transfer of ML-MoS_2_ films from sapphire to target substrates were assisted by wet etching in KOH solution (1 Mol/L, 110 °C). The process is illustrated in Supplementary Fig. 3. Au contact electrodes (10–15 nm) with clean interfaces were fabricated by e-beam evaporation with an ultra-low deposition rate of ~0.01 Å·s^−1^. All the electrical measurements were carried out in a Janis probe station at a base pressure of 10^−6^ Torr with an Agilent semiconductor parameter analyzer (B1500, high resolution modules) and Agilent digital oscilloscope (DSO-X 3054 A) at room temperature.

### STEM characterizations

The cross-sectional STEM characterizations were carried out by an aberration corrected JEOL ARM 300 F transmission electron microscope operated at 300 kV.

## Supplementary information

Supplementary Information

Peer Review File

Description of Additional Supplementary Files

Supplementary Movie 1

## Data Availability

The Source Data underlying the figures of this study are available at 10.6084/m9.figshare.22776989. All raw data generated during the current study are available from the corresponding authors upon request.
