# Supplementary material for: Low power flexible monolayer MoS2 integrated circuits
Source: Nat Commun. 2023 Jun 19;14:3633. doi: 10.1038/s41467-023-39390-9 (PMC10279675; doi:10.1038/s41467-023-39390-9)
Supplement: Supplementary file 1 — Supplementary Information [file 41467_2023_39390_MOESM1_ESM.pdf]

Supplementary Information for

Low power flexible monolayer MoS_2 integrated circuits

Jian Tang[1,2,†], Qinqin Wang[1,2,†], Jinpeng Tian[1,2,†], Xiaomei Li[1,2,3], Na Li[1,4], Yalin Peng[1,2], Xiuzhen Li[1,2], Yanchong Zhao[1,2], Congli He[5], Shuyu Wu[6], Jiawei Li[1,2], Yutuo Guo[1,2], Biying Huang[1,2], Yanbang Chu[1,2], Yiru Ji[1,2], Dashan Shang[6], Luojun Du[1,2], Rong Yang[1,4], Wei Yang[1,2,4], Xuedong Bai[1,2], Dongxia Shi[1,2] & Guangyu Zhang[1,2,4*]

[1]*Beijing National Laboratory for Condensed Matter Physics and Institute of Physics, Chinese Academy of Sciences, Beijing 100190, China*
[2]*School of Physical Sciences, University of Chinese Academy of Sciences, Beijing 100190, China*
[3]*Shanghai Key Laboratory of Multidimensional Information Processing, East China Normal University, Shanghai, China*
[4]*Songshan Lake Materials Laboratory, Dongguan 523808, China*
[5]*Institute of Advanced Materials, Beijing Normal University, Beijing 100875, China*
[6]*Institute of Microelectronics, Chinese Academy of Sciences, Beijing 100029, China*

[†]These authors contributed equally to this work.
*Corresponding author, e-mail: gyzhang@iphy.ac.cn

Supplementary Figures

Supplementary Figure 1. The HfO_2 dielectric deposition, surface morphology characterizations and leakage current measurements.
Supplementary Figure 2. Characterizations of high-quality monolayer ML-MoS_2 films with large domain sizes.
Supplementary Figure 3. Batch fabrications of Wafer-scale ML-MoS_2 TFTs.
Supplementary Figure 4. The capacitance/dielectric constant measurements of HfO_2 films.
Supplementary Figure 5. The AFM image of the PET substrate.
Supplementary Figure 6. The carrier densities extraction based on the C-V measurement of MOSFET.
Supplementary Figure 7. Electrical properties of L_{ch}=150/300 nm ML-MoS_2 TFTs (on SiO_2) with 5-nm HfO_2 as the dielectric layer.
Supplementary Figure 8. The electrical properties comparisons of MoS_2 FETs with 5 nm HfO_2, 10 nm HfO_2, and 30 nm Al_2O_3 as dielectric layer.
Supplementary Figure 9. The leakage current characterizations of flexible MoS_2 FETs.
Supplementary Figure 10. The extraction of contact resistance based on short channel devices.
Supplementary Figure 11. Output properties of flexible ML-MoS_2 inverters.
Supplementary Figure 12. Characterizations of the unit TFT in an inverter.
Supplementary Figure 13. The oscillating frequency of 3-stage RO by reducing the parasitic capacitances from the contact electrodes/additional outer MoS_2 region.
Supplementary Figure 14. The strain measurement set up.
Supplementary Figure 15. The strain analysis based on Raman characterizations of monolayer MoS_2 on PET substrate.
Supplementary Figure 16. Bending test of flexible ML-MoS_2 TFTs and 5-stage RO.
Supplementary Figure 17. The leakage current analysis with strain.

Supplementary Tables

Supplementary Table 1. The comparison of the MoS_2 growth and device benchmarks.
Supplementary Table 2. The device parameter comparisons of ML-MoS_2 FETs on rigid substrate.
Supplementary Table 3. Comparisons of flexible ML-MoS_2 devices.
Supplementary Table 4. Voltage gains of logic inverter.
Supplementary Table 5. Power consumption comparisons of low-power inverters.
Supplementary Table 6. Propagation stage delay versus supply voltage of ROs.

Supplementary Notes

Supplementary Note 1. The optimization routes of MoS_2 film quality.
Supplementary Note 2. The parasitic capacitance optimizations of MoS_2 ring oscillator.
Supplementary Note 3. The strain analysis based on Raman characterizations.

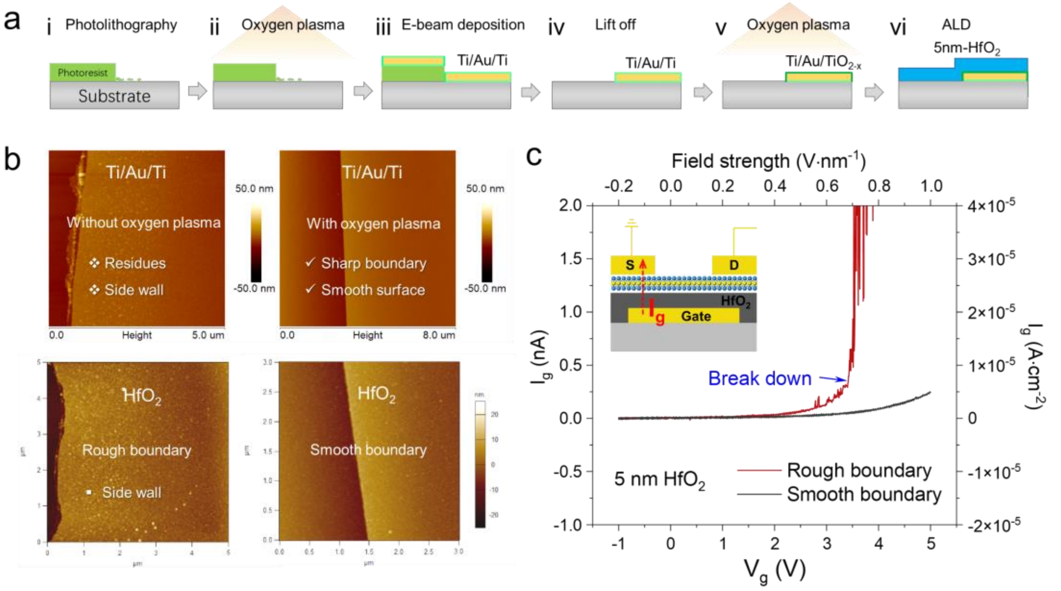

Supplementary Figure 1. The HfO_2 dielectric deposition, surface morphology characterizations and leakage current measurements. a. The schematic illustration of highly dense HfO_2 deposition process on back-gate electrode. **b.** Typical AFM height images of fabricated Ti/Au/Ti back-gate electrode with/without plasma cleaning photoresist residues, and the HfO_2 morphology characterizations after deposited on Ti/Au/TiO_{2-x} back-gate electrode with rough/smooth boundary. **c.** The leakage current measurements of 5-nm HfO_2 on Ti/Au/TiO_{2-x} metal gates with rough (red line) and smooth boundary (black line). The breakdown voltage could exceed 5 V for the smooth boundary samples.

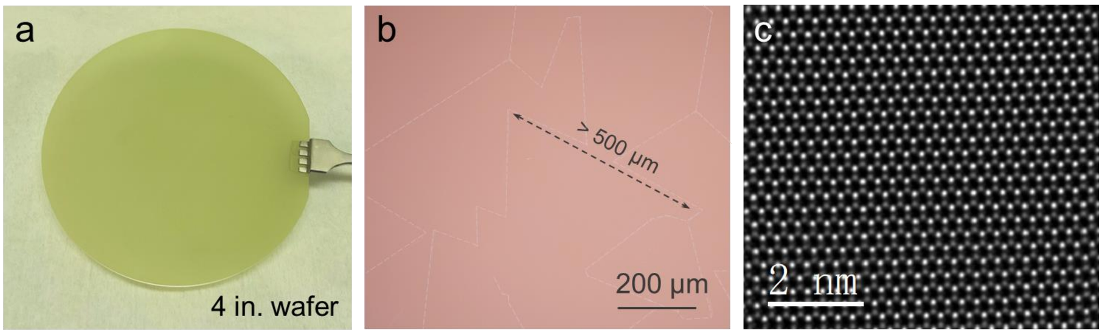

Supplementary Figure 2. Characterizations of high-quality monolayer ML-MoS_2 films with large domain sizes. a. Optical image of a 4-inch ML-MoS_2 wafer. **b.** Optical image of ML-MoS_2 films with well-stitched domains of 200-500 μm in size and 100 % coverage. Scale bar, 200 μm. **c.** High-resolution atomic structure of the MoS_2 film suggests the high crystallinity quality. Scale bar, 2 nm.

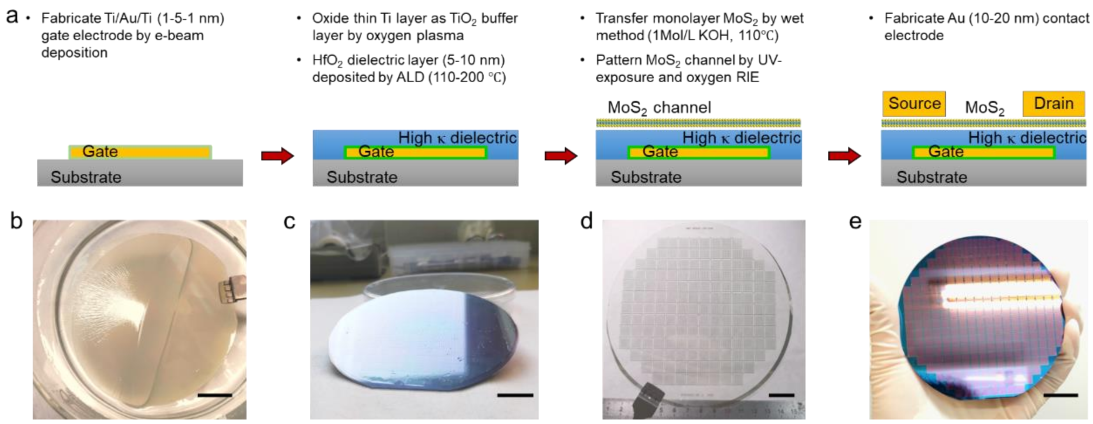

Supplementary Figure 3. Batch fabrications of Wafer-scale ML-MoS_2 TFTs. a. Schematic illustration of the device's fabrication process. **b.** Floating of a 4-inch MoS_2 film supported by the PMMA layer in deionized water. **c.** Transfer of the MoS_2/PMMA film onto as-prepared buried-gate electrodes. The transferred PMMA/MoS_2 on substrate is intact and smooth without wrinkles, bubbles, or any damages. **d-e.** Optical image of 4-inch scale ML-MoS_2 TFTs on the **(d)** PET substrate or **(e)** SiO_2 substrate. Scale bars in **b-e**, 2 cm.

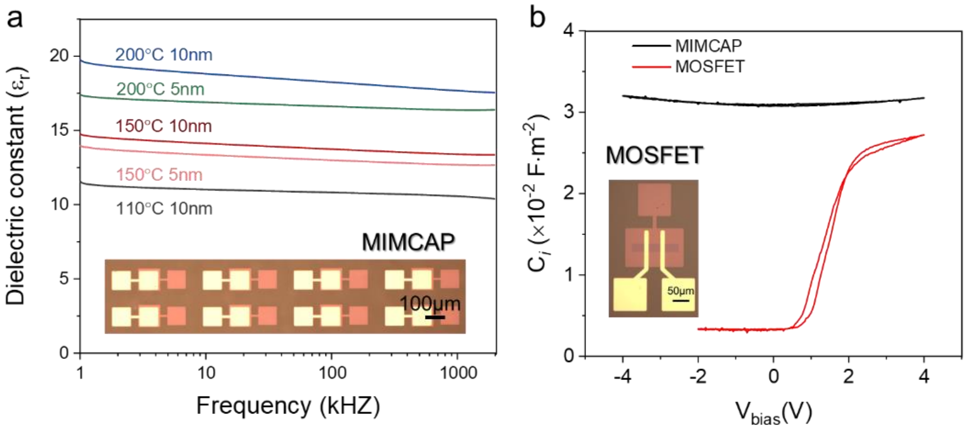

Supplementary Figure 4. The capacitance/dielectric constant measurements of HfO_2 films. a. Measurements of device capacitance/dielectric constant as a function of frequency of HfO_2 dielectric layers with different thicknesses deposited at various temperatures. Inset illustrates the optical image of the HfO_2 capacitance devices based on MIM structure. **b.** Capacitivity-voltage (C-V) measurements comparisons of 5 nm HfO_2 with a frequency of 100 KHZ based on MOS and MIM structure.

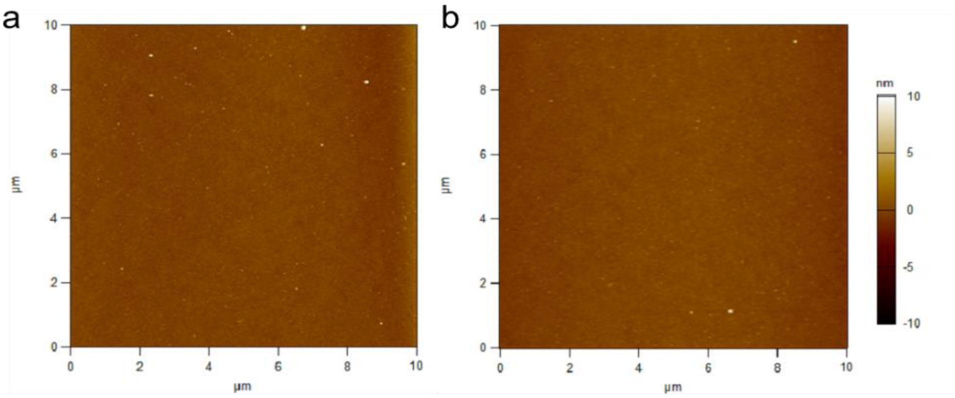

Supplementary Figure 5. The AFM image of the PET substrate. The surface roughness is ~0.72 ± 0.1 nm.

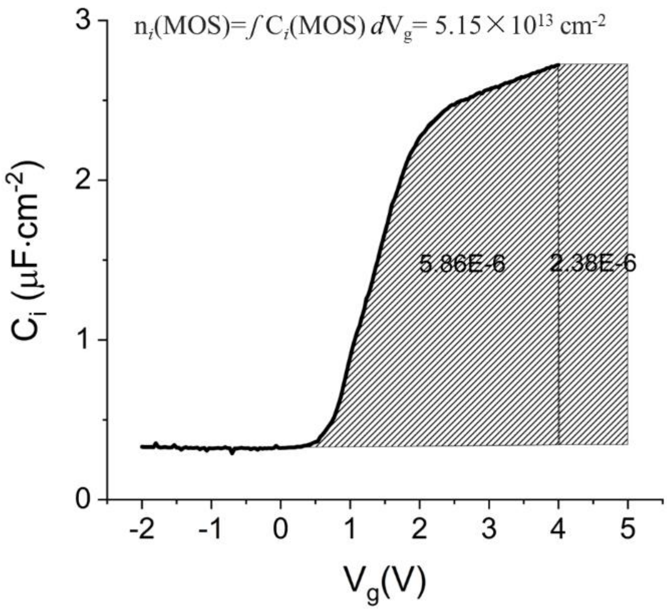

Supplementary Figure 6. The carrier densities extraction based on the C-V measurement of MOSFET. The total carrier densities in MoS_2 were integrated through the shadow region.

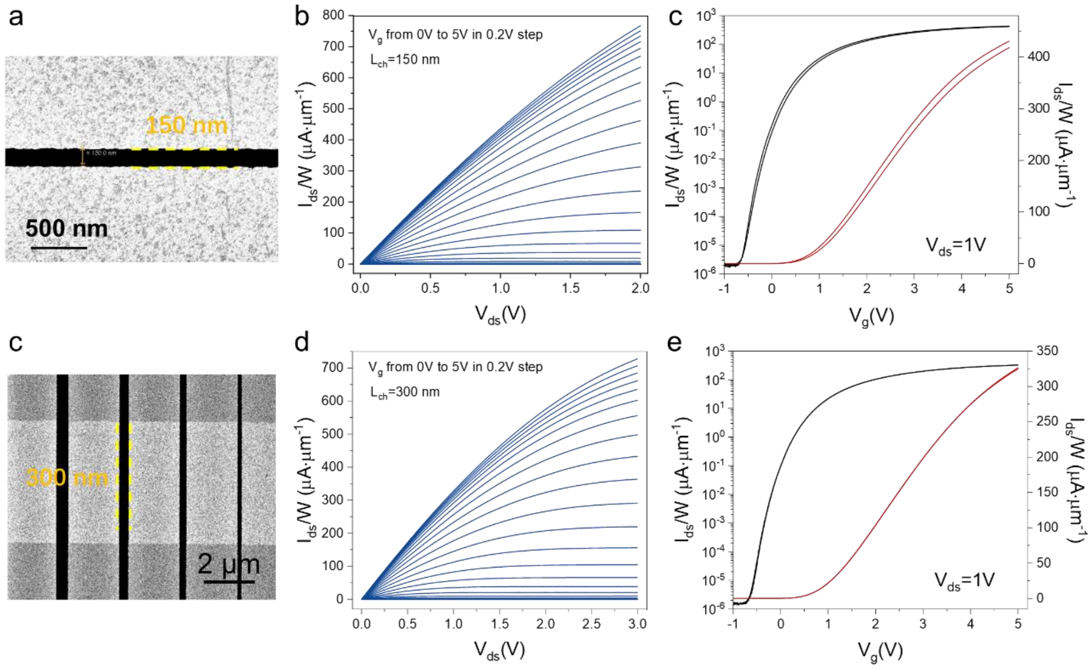

Supplementary Figure 7. Electrical properties of L_{ch}=150/300 nm ML-MoS_2 TFTs (on SiO_2) with 5-nm HfO_2 as the dielectric layer. a/c. SEM image of the device channel region. **b-c/d-e.** Corresponding output and transfer curves of ML-MoS_2 TFTs with L_{ch}=150/300 nm. The on-state current density is 436/328 μA·μm^{-1} at V_{ds}=1 V, and the maximum current density could approach 768/727 μA·μm^{-1} at V_{ds}=2/3 V. The on/off ratio is over 10^8.

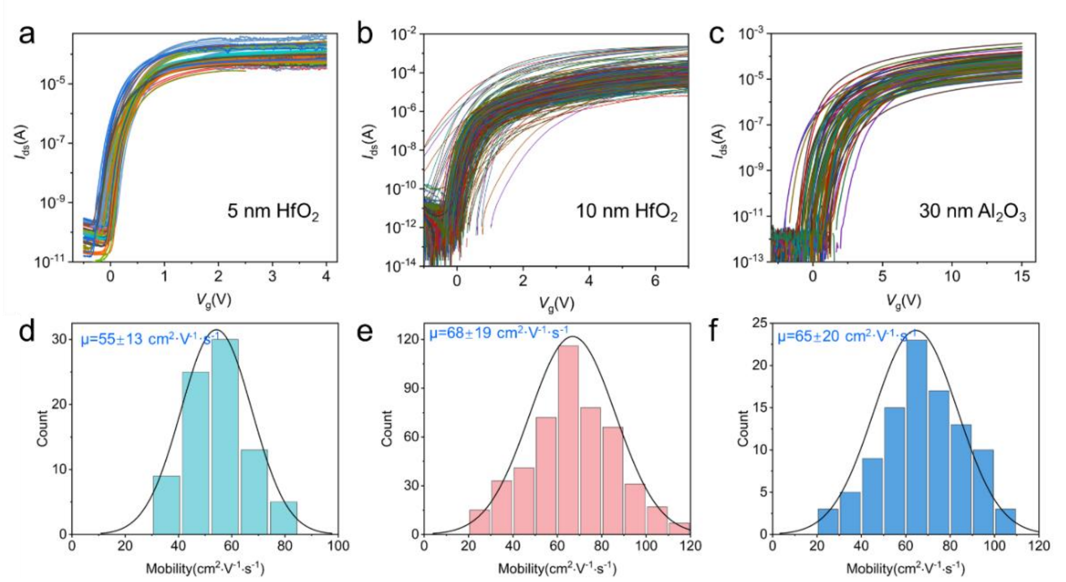

Supplementary Figure 8. The electrical properties comparisons of MoS_2 FETs with 5 nm HfO_2, 10 nm HfO_2, and 30 nm Al_2O_3 as dielectric layer. a-c. Transfer curves of MoS_2 FET with V_{ds}=1V. **d-f.** Corresponding device mobility distribution. The average device mobility of 5 nm HfO_2 is 55±13 $cm^2·V^{-1}·s^{-1}$, 10 nm HfO_2 is 68±19 $cm^2·V^{-1}·s^{-1}$ and 30 nm Al_2O_3 is 65±20 $cm^2·V^{-1}·s^{-1}$.

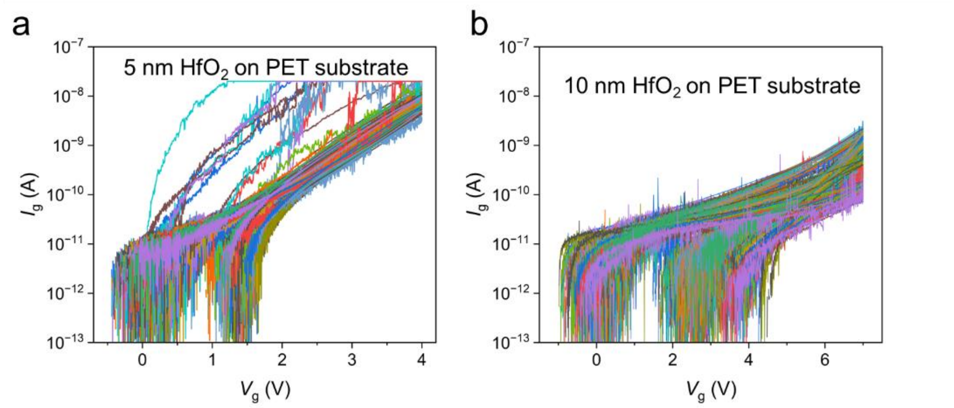

Supplementary Figure 9. The leakage current characterizations of flexible MoS_2 FETs. a. The leakage current of MoS_2 FETs based on 5 nm HfO_2 on PET substrate. **b.** The leakage current of MoS_2 FETs based on 10 nm HfO_2 on PET substrate.

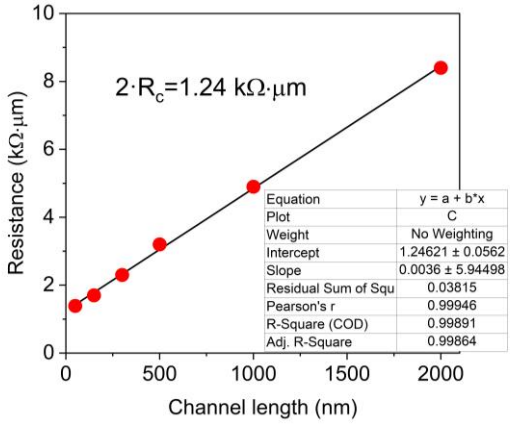

Supplementary Figure 10. The extraction of contact resistance based on short channel devices. The R_c extraction is based on the device with L_{ch} from 50 nm to 2 μm. The extracted R_c is around 0.62 kΩ·μm, which is very close to value extracted from the long channel devices of 0.59 kΩ·μm in Fig. 2d based on long channel devices in main text.

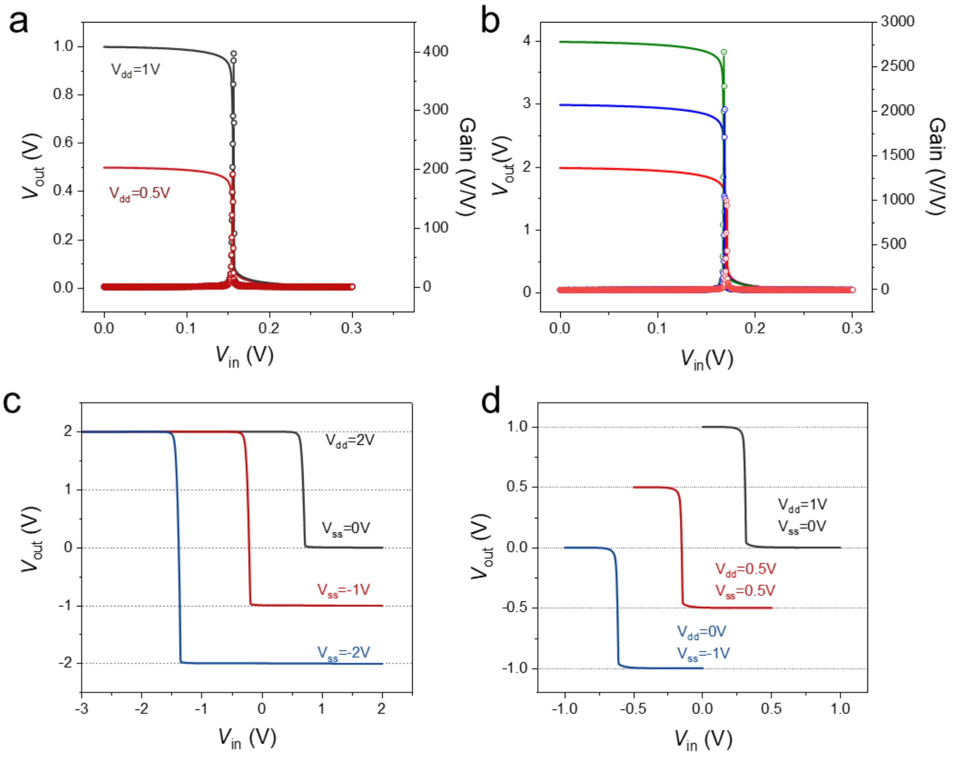

Supplementary Figure 11. Output properties of flexible ML-MoS_2 inverters. a-b. Output curves and corresponding gain values of an inverter. The maximum inverter gain is 2670 at V_{dd}=4V. **c.** Swing threshold voltages of the inverter shift negatively with decreasing the operation voltages of the V_{ss} terminal. **d.** Swing threshold voltages shift negatively with synchronously decreasing the V_{ss} and V_{dd} voltages.

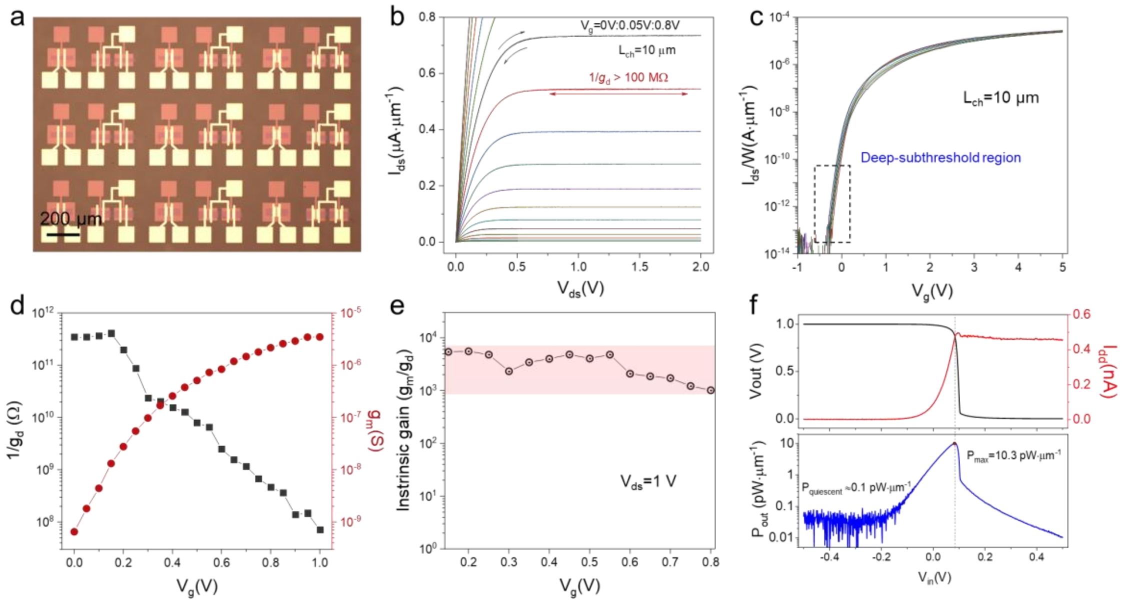

Supplementary Figure 12. Characterizations of the unit TFT in an inverter. a. Optical image of the ML-MoS_2 TFT and inverter array with the same device geometry. **b.** Output curves of a unit TFT with L_{ch}=10 μm and W=40 μm. **c.** Corresponding transfer curves of the device. **d.** Extracted values of $1/g_d$ and g_m as a function of V_g at V_{ds}=1V, where g_d is output conductance and g_m is transconductance. **e.** Intrinsic gain (g_m/g_d) as a function of V_g. **f.** Output voltages (V_{out}) of the ML-MoS_2 inverter, channel currents (I_{dd}) and corresponding output-power consumptions (P_{out}) as a function of input voltages (V_{in}) (also see Supplementary Table 5 for power consumption comparisons).

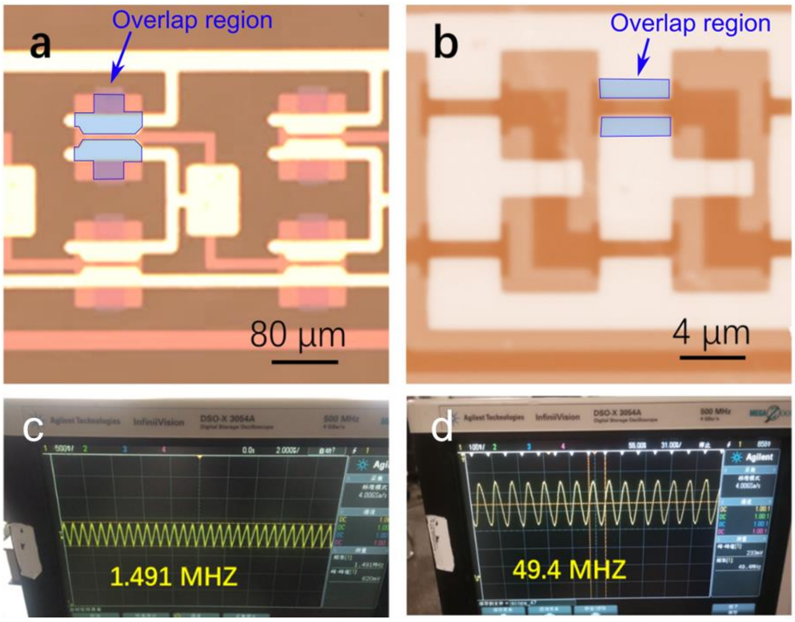

Supplementary Figure 13. The oscillating frequency of 3-stage RO by reducing the parasitic capacitances from the contact electrodes/additional outer MoS_2 region. a-b. the device design optimization of ring oscillators. **c-d.** The output oscillating signals of ROs after optimizing the device design. Corresponding state delay is 111.8 ns for (**a**) and 3.4 ns for (**b**), individually.

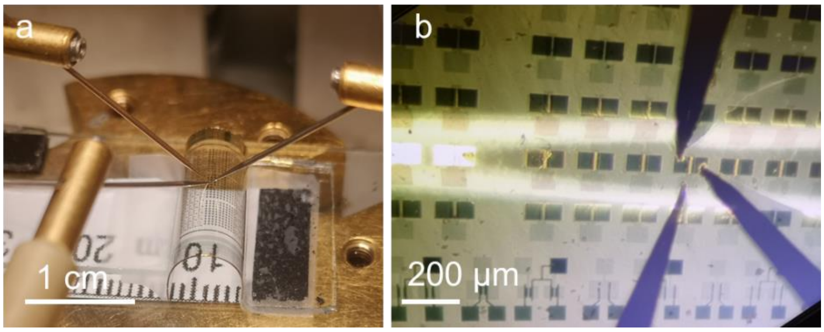

Supplementary Figure 14. The strain measurement set up. a. The optical image of the set up for strain measurements. **b.** Optical image of the device with the bending radius *R*=3 mm.

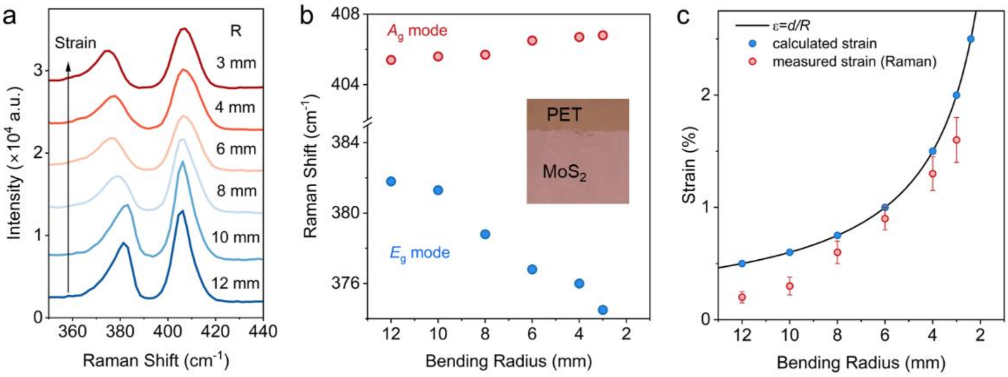

Supplementary Figure 15. The strain analysis based on Raman characterizations of monolayer MoS_2 on PET substrate. a. Raman spectrum of monolayer MoS_2 with bending radius from 12 mm to 3 mm. **b.** The statistical E_g and A_g mode position as a function of bending radius. **c.** The comparison between calculated strain ($\varepsilon=d/R$, where 2d is the thickness of the PET substrate) and measured strain based on Raman results. Error bars are taken from strain error. Note that the pristine Raman mode position was celebrated by the monolayer MoS_2 without strain.

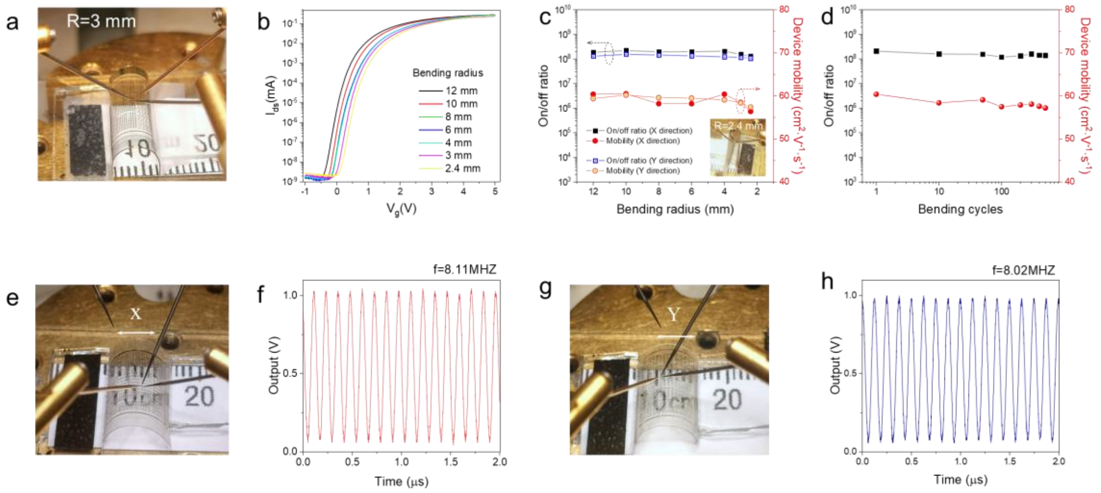

Supplementary Figure 16. Bending test of flexible ML-MoS_2 TFTs and 5-stage RO. a. Optical photograph of the bending test setup and a sample at a bending radius of R=3 mm. **b.** Transfer curves of a ML-MoS_2 TFT with bending radius varying from 12 to 2.4 mm. **c.** Dependence of the On/Off ratio and device mobility on the bending radius along the X-/Y- direction. Inset to (**c**) shows the bending radius at R=2.4 mm. **d.** Variations of the On/Off ratio and device mobility as a function of bending cycles with bending radius of R=3 mm. Note that the bending radius of R=3 mm corresponds to the strain of ε=1.6 ±0.2 % as verified by Raman spectrum. **e/g.** Optical images of a flexible RO at the bending radius of R=4 mm along X-/Y-direction. **f/h.** The output signal of the RO at V_{dd}=3 V with f=8.11/8.02 MHZ.

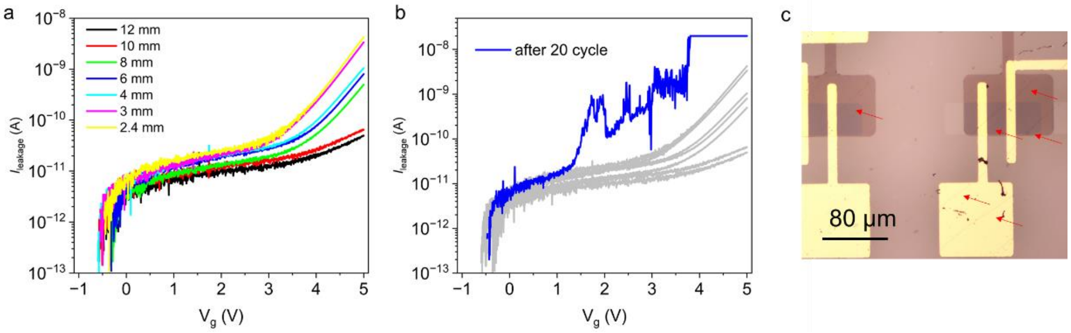

Supplementary Figure 17. The leakage current analysis with strain. a. The leakage current recorded during the bending test with the bending radius from 12 mm to 2.4 mm. **b.** The leakage current increases over than the compliance setting of ~20 nA after bending 20 times. **c.** The optical image of the device after bending 20 times, where the red arrows indicate the tiny wrinkles.

Supplementary Table 1. The comparison of the MoS_2 growth and device benchmarks.

**Refs**	**Domain size**	**Orientation**	**Device mobility** $cm^2 \cdot V^{-1} \cdot s^{-1}$
Ref [1]. Polycrystalline MoS_2 on SiO_2	<1 μm	>2	7
Ref [2]. Polycrystalline MoS_2 on fused silica	<1 μm	>2	30
Ref [3]. MoS_2 epitaxy on sapphire	2-5 μm	2	40
Ref [4]. MoS_2 on glass	400 μm	>2	11
Ref [5]. MoS_2 epitaxy on Au	Single domain	1	10
Ref [6]. MoS_2 epitaxy on sapphire	180 μm	2	70
Ref [7]. MoS_2 on sapphire	Single domain	1	80
Ref [8]. MoS_2 on HfO_2	Single domain	1	62
This work	200-500μm	2	70

Supplementary Table 2. The device parameter comparisons of ML-MoS_2 FETs on rigid substrate.

Refs	T_{ox}	EOT (nm)	L_{ch} (nm)	I_{on} @V_{ds}=1V (μA/μm)	I_{on_max} (μA/μm)	SS (mV/dec)	Contact
Ref [9]	300 nm SiO_2	300	10	190	550 (V_{ds}=3V)	/	Graphene
Ref [10]	6 nm HfO_2	2	8.2	2.5	/	140	Au
Ref [11]	10 nm HfO_2	3.3	15	5	/	75	Graphene
Ref [12]	100 nm SiN_x	56.7	35	840	1135 (V_{ds}=1.5V)	/	Bi (R_c=123Ω·μm)
	100 nm SiN_x	56.7	120	390	560 (V_{ds}=1.5V)	/	Bi
Ref [13]	5 nm HfO_2	1.5	50	156	250 (V_{ds}=1.6V)	73	Ni/Au (R_c=2.3kΩ·μm)
Ref [14]	30 nm SiO_2	30	72	280	320 (V_{ds}=2V)	900	Ag/Au (R_c=1.3kΩ·μm)
	30 nm SiO_2	30	4.9×10^3	24	240 (V_{ds}=20V)	/	/
Ref [15]	6 nm Al_2O_3	/	80	20	/	180	Au
Ref [16]	30 nm SiO_2	30	400	280	700 (V_{ds}=5V)	1000	Au (R_c=0.48kΩ·μm)
Ref [17]	93 nm SiO_2	93	450	140	240 (V_{ds}=2V)	1600	Ag/Au (R_c=0.18kΩ·μm)
Ref [18]	30 nm HfO_2	/	500	100	320 (V_{ds}=4.2V)	178	Au
Ref [7]	30 nm Al_2O_3	19.2	500	140	450 (V_{ds}=6V)	210	Au
Ref [19]	72 nm Al_2O_3	/	2×10^3	20	160 (V_{ds}=8V)	/	Mo/Au
Ref [20]	15 nm hBN	16.2	1.9×10^3	11	44 (V_{ds}=4V)	64	Pd/Au (R_c=3.5kΩ·μm)
Ref [1]	300 nm SiO_2	300	2×10^4	0.14	/	/	Ti/Au
Ref [21]	270 nm SiO_2	270	3×10^4	0.05	1.3 (V_{ds}=40V)	/	Ti/Au
Ref [22]	285 nm SiO_2	285	10^5	0.08	0.4 (V_{ds}=5V)	/	Ti/Au

Notes: All the carrier densities are calculated based on the MIM capacitance model.

Supplementary Table 3. Comparisons of flexible ML-MoS_2 devices[23,24].

Parameters	NE 2021, 4, 495	NE 2020, 3, 711 (Our Previous work)	This work
Channel material	1.8 cm MoS_2 films grown on SiO_2 substrate	4-inch ML-MoS_2 average domain size: ~20 μm	4-inch ML-MoS_2 average domain size: >200 μm
High-κ dielectric layer	35 nm Al_2O_3 EOT~16.4 nm	30~80 nm Al_2O_3 EOT~15-42 nm	5~10 nm HfO_2 EOT~1-2 nm
Maximum carrier densities (n_i) in MoS_2	~1.575×10^{13} cm^{-2} (MOSCAP)	~2.4×10^{13} cm^{-2} (MIMCAP)	~5.15×10^{13} cm^{-2} (MOSCAP) ~9.656×10^{13} cm^{-2} (MIMCAP)
Device structure	Local top-gate	Global back-gate	Local back-gate
Contact resistance (R_c)	Au contact 0.25-2.3 kΩ·μm	Au/Ti-Au contact 2.9 kΩ·μm @ $n_i \approx$ 2.4×10^{13} cm^{-2}	Au contact 0.59 kΩ·μm @ $n_i \approx 2.8\times10^{13}$ cm^{-2}
Maximum current densities	466 $\mu A\cdot\mu m^{-1}$ @V_g =16 V,V_{ds}=1 V, L_{ch}=82 nm	35 $\mu A\cdot\mu m^{-1}$ @V_g =80 V, V_{ds}=5 V, L_{ch}= 6 μm	53.8 $\mu A\cdot\mu m^{-1}$ @V_g =7 V, V_{ds}=1 V, L_{ch}= 5 μm
ON/OFF ratio	2×10^6	10^9-10^{10}	5×10^7
Average mobility	27~30 $cm^2\cdot V^{-1}\cdot s^{-1}$	~55 $cm^2\cdot V^{-1}\cdot s^{-1}$	~70 $cm^2\cdot V^{-1}\cdot s^{-1}$
Subthreshold swings (SS)	730~1000 $mV\cdot dec^{-1}$	~1000 $mV\cdot dec^{-1}$	83 $mV\cdot dec^{-1}$
Pinch-off voltage distribution	-12~8 V	0~5 V	-0.4 ~ +0.4 V
Inverter gain	4-7 @ V_{dd}=3 V	43 @ V_{dd}=2 V 107 @ V_{dd}=4 V	120@V_{dd}=0.5V 2670 @ V_{dd}=4 V
Minimum supply voltage	N/A	V_{dd}=2 V	V_{dd}=0.5 V
Ring oscillator	N/A	Start to oscillate @ V_{dd}=5 V f_{max}=13.12 MHZ @ V_{dd}=15 V	Start to oscillate @ V_{dd}=0.3 V f_{max}=24.8 MHZ @ V_{dd}=5 V
TFT Power	N/A	400 $pW\cdot\mu m^{-1}$	10.3 $pW\cdot\mu m^{-1}$

Supplementary Table 4. Voltage gains of logic inverter.

Refs	Channel materials	Dielectric layer	V_{dd} (V)	Gain	Substrate
Ref [24]	MoS_2	Al_2O_3	2/4	43/107	Flexible
Ref [25]	MoS_2/WSe_2	Ion-gel	2	110	Flexible
Ref [26]	CNT	Al_2O_3	3	25	Flexible
Ref [27]	CNT/IZGO	Al_2O_3-SiO_2	6	20.9	Flexible
Ref [28]	CNT	Al_2O_3	2	290	Flexible
Ref [29]	CNT	Al_2O_3	5	16	Flexible
Ref [30]	CNT	HfO_2	2	30	Flexible
Ref [31]	CNT	HfO_2	2	46.6	Flexible
Ref [32]	Te film	ZrO_2	1/2	22/38	Flexible
Ref [33]	ZnO	Al_2O_3	5	130	Flexible
Ref [34]	C8-BTBT	PVC	2	260	Flexible
Ref [35]	MoS_2	Al_2O_3	5	20	Rigid
Ref [36]	MoS_2	ZrO_2	4	70	Rigid
Ref [37]	MoS_2	HfO_2	2/6/10	10/110/155	Rigid
Ref [38]	MoS_2	Al_2O_3	5	60	Rigid
Ref [39]	MoS_2	HfO_2	3	23	Rigid
Ref [40]	MoS_2	hBN	5	90	Rigid
Ref [41]	MoS_2	HfO_2	2	5	Rigid
Ref [42]	MoS_2	HfO_2	5	16	Rigid
Ref [43]	MoS_2/BP	High-κ	3	152	Rigid
Ref [44]	MoS_2/CNT	SiO_2	2/5	7/15	Rigid
Ref [45]	WSe_2/MoS_2	HfO_2	0.5	12	Rigid
Ref [46]	$MoS_2/MoTe_2$	Al_2O_3	0.5/1	7.7/33.3	Rigid
Ref [47]	$MoTe_2$	HfO_2	2/6	16/35	Rigid
Ref [48]	WSe_2	SiO_2	2.5/5.5	135/340	Rigid
Ref [49]	WSe_2	ZrO_2	3	12	Rigid
Ref [50]	BP	HfO_2	1/2	9/13	Rigid
Ref [51]	BP	Al_2O_3	0.2/0.4/0.6/0.8/1	6.4/15/21.5/24/33.3	Rigid
Ref [52]	WSe_2	Al_2O_3	2	37	Rigid
This work	MoS_2	HfO_2	0.5/1/2/4	120(192)/397/1000/2670	Flexible

Supplementary Table 5. Power consumption comparisons of low-power inverters[34,53].

Refs	Power supply voltages	Power consumption
IGZO inverter [34]	V_{dd}=2V (Gain 260)	~620 pW
Organic (C8-BTBT/PS) inverter [53]	V_{dd}=2V (Gain ~210)	~140 pW
This work (MoS_2 inverter)	V_{dd}=0.5V (Gain 120)	408 pW (10.3 pW·μm^{-1})

Supplementary Table 6. Propagation stage delay versus supply voltage of ROs.

Refs	Channel materials	Stage number	V_{dd}(V)	Propagation stage delay τ_{pd} (s)
Ref [24]	MoS_2	5	5~15	1.2×10^{-7}~7.6×10^{-9}
Ref [31]	CNT	15	2	2.27×10^{-5}
Ref [54]	CNT	7/15	3~15	2.7×10^{-4}~6.2×10^{-6}
Ref [26]	CNT	5	3~17	2.5×10^{-7}~5.7×10^{-9}
Ref [55]	CNT	5	1.4~2.5	4.35×10^{-5}~5.26×10^{-5}
Ref [56]	CNT	5	2.5~8	2.5×10^{-10}~9.5×10^{-8}
Ref [27]	CNT/IZGO	51~501	6	5×10^{-6}~2.17×10^{-6}
Ref [57]	IGZO	7	5~15	1.59×10^{-6}~2.1×10^{-7}
Ref [58]	ZnO	15	2~18	3.05×10^{-6}~1.6×10^{-8}
Ref [59]	DNTT	5	2.1~5	6.2×10^{-5}~1.8×10^{-5}
Ref [60]	DNTT	11	1.5~4.4	1.43×10^{-7}~7.9×10^{-8}
This work	MoS_2	3-11	0.3~5	7.8×10^{-8}~6.8×10^{-9}

Supplementary Note 1. The optimization routes of MoS_2 film quality

To gain better MoS_2 film quality for large-scale electronic applications, there are two important challenges below:

First, **the grain size is a key factor for device performance**. The comparisons in Table S1 indicate that the grain size plays a key role in the final device mobility. We know that the domain boundary would scatter electrons and thus reduce the device mobility to some extent, especially for the small grain size films with higher domain boundary densities. Thus, we may be able to enhance device mobility and ultimately improve device performance through enlarging the grain size and controlling the orientation of the domain. Supplementary Figure 2 a-b shows an optical image of high-quality 4-inch ML-MoS_2 wafer with large domain sizes between 200 μm to 500 μm. These domains are well-stitched together to form a continuous film with 100 % coverage. Related growth details could refer to

our work entitled ‘Wafer-scale highly oriented monolayer MoS_2 with large domain sizes’ on *Nano Letters* (2020)[6].

Next, **controlling the defect densities is an effective way to enhance the film electronic quality.** In order to identify the local defects in our monolayer MoS_2 samples, we have characterized our samples by TEM. Typical result is shown in Supplementary Figure 2c, and we didn’t see high densities of defects based on this local characterization. Thus, in order to investigate the defect densities at the macro scale, we developed a wet etching method to visualize those structural defects. Please refer to our work entitled ‘Direct visualization of structural defects in 2D semiconductor’ on *Chin. Phys. B* (2022)[61] for details. We have compared three types of samples, i.e., exfoliated MoS_2 flakes, small-grained (~few µm) CVD MoS_2 films, and large-grained (~few hundreds of µm, the samples used in this work) CVD MoS_2 films. Our statistical results reveal that the large-grained samples have much lower density of etched pits than the exfoliated samples (by 1 order of magnitude) and small-grained samples (by 2-3 order of magnitudes). The defect density characterizations tell us that the large-grained MoS_2 samples have the lowest density of both point defects and grain boundaries, providing the highest quality.

Supplementary Note 2. The parasitic capacitance optimizations of MoS_2 ring oscillator

We optimized the device structure of MoS_2 ring oscillator to reduce the impact of parasitic capacitance on the frequency as shown in Supplementary Figure 13.

For the device in Supplementary Figure 13a, the channel length and channel width are L=5 µm, w=40 µm, individually. And the parasitic capacitance is roughly:

$$C_p(a)=2\times(C_{contact}+C_{MoS2\_region})$$

$$=2\times[10\ \mu m\times(80 + 70)\ \mu m\times2\times C_{i\_metal} + (100 - 45)\ \mu m\times40\ \mu m\times C_{i\_MoS2}]=131.8\ pF$$

For the device in Supplementary Figure 13b, the channel length and channel width are L=1 µm, w=3 µm, individually. And the parasitic capacitance is greatly reduced:

$$C_p(b)=2\times C_{contact}=2\times(1\ \mu m\times4\ \mu m\times2\times C_{i\_metal}) = 0.205\ pF$$

The large parasitic capacitances in device (**a)** mainly come from the contact region/additional outer MoS_2 region (as outlined by the blue shadow), while the parasitic capacitance/per stage in device **(b)** is small due to the reduced overlapped region and the eliminated additional outer MoS_2 region (as outlined by the blue shadow). As a result, the corresponding experimentally achieved output frequencies are 1.491 MHZ (a) and 49.4 MHZ (b).

Supplementary Note 3. The strain analysis based on Raman characterizations.

We carry out Raman measurements to check whether the strain is applied correctly as shown in Supplementary Figure 15. The E_g mode has a red shift from ~382 cm^{-1} to 374 cm^{-1} with the bending radius changing from 12 mm to 3 mm, while the A_g mode positions roughly unchanged around 406 cm^{-1}. Based on the reference work[61], we could calculate the strain we applied on monolayer MoS_2 roughly changed from 0.2% to 1.6%, which is a little smaller than the expected values from 0.5% to 2%, but follows well with the trend of the calculated strain based on formula $\varepsilon=d/R$.

Supplementary References

1 Zhang, J. *et al.* Scalable growth of high-quality polycrystalline MoS_2 monolayers on SiO_2 with tunable grain sizes. *ACS Nano* **8**, 6024-6030 (2014).
2 Kang, K. *et al.* High-mobility three-atom-thick semiconducting films with wafer-scale homogeneity. *Nature* **520**, 656-660 (2015).
3 Yu, H. et al. Wafer-scale growth and transfer of highly oriented monolayer MoS_2 continuous films. *ACS nano* **11**, 12001-12007 (2017).
4 Yang, P. *et al.* Batch production of 6-inch uniform monolayer molybdenum disulfide catalyzed by sodium in glass. *Nat. Commun.* **9**, 979 (2018).
5 Yang, P. *et al.* Epitaxial growth of centimeter-scale single-crystal MoS_2 monolayer on Au (111). *ACS nano* **14**, 5036-5045 (2020).
6 Wang, Q. *et al.* Wafer-scale highly oriented monolayer MoS_2 with large domain sizes. *Nano Letters* **20**, 7193-7199 (2020).
7 Li, T. *et al.* Epitaxial growth of wafer-scale molybdenum disulfide semiconductor single crystals on sapphire. *Nat. Nanotechnol.* (2021).
8 Kim, K. *et al.* Non-epitaxial single-crystal 2D material growth by geometric confinement. *Nature* 1-7 (2023).
9 Xie, L. et al. Graphene-Contacted Ultrashort Channel Monolayer MoS_2 Transistors. *Adv. Mater.* **29**, 02522 (2017).
10 Xu, K. *et al.* Sub-10 nm Nanopattern Architecture for 2D Material Field-Effect Transistors. *Nano Lett.* **17**, 1065-1070 (2017).
11 Nourbakhsh, A. *et al.* 15-nm Channel Length MoS_2 FETs with Single- and Double-Gate structures. *2015 Symposium on Vlsi Technology (Vlsi Technology)* (2015).
12 Shen, P. C. *et al.* Ultralow contact resistance between semimetal and monolayer semiconductors. *Nature* **593**, 211-217 (2021).
13 Zhu, Y. *et al.* Monolayer Molybdenum Disulfide Transistors with Single-Atom-Thick Gates. *Nano Lett.* **18**, 3807-3813 (2018).
14 Smithe, K. K. H. *et al.* High-Field Transport and Velocity Saturation in Synthetic Monolayer MoS_2. *Nano Lett.* **18**, 4516-4522 (2018).
15 Cao, W., Liu, W., Kang, J. & Banerjee, K. An Ultra-Short Channel Monolayer MoS_2 FET Defined By the Curvature of a Thin Nanowire. *IEEE Electron Device Letters* **37**, 1497-1500 (2016).
16 McClellan, C. J., Yalon, E., Smithe, K. K. H., Suryavanshi, S. V. & Pop, E. High Current Density in Monolayer MoS_2 Doped by AlO_x. *Acs Nano* **15**, 1587-1596 (2021).
17 Rai, A. *et al.* Air Stable Doping and Intrinsic Mobility Enhancement in Monolayer Molybdenum Disulfide by Amorphous Titanium Suboxide Encapsulation. *Nano Lett.* **15**, 4329-4336 (2015).
18 Lembke, D. & Kis, A. Breakdown of high-performance monolayer MoS_2 transistors. *ACS Nano* **6**, 10070-10075 (2012).
19 Kang, J., Liu, W. & Banerjee, K. High-performance MoS_2 transistors with low-resistance molybdenum contacts. *Appl. Phys. Lett.* **104** (2014).
20 Zheng, X. *et al.* Patterning metal contacts on monolayer MoS_2 with vanishing Schottky barriers using thermal nanolithography. *Nat. Electron.* **2**, 17-25 (2019).
21 Park, W. *et al.* Photoelectron spectroscopic imaging and device applications of large-area patternable single-layer MoS_2 synthesized by chemical vapor deposition. *ACS Nano* **8**, 4961-4968 (2014).
22 Najmaei, S. *et al.* Vapour phase growth and grain boundary structure of molybdenum disulphide atomic layers. *Nat. Mater.* **12**, 754-759 (2013).
23 Daus, A. *et al.* High-performance flexible nanoscale transistors based on transition metal dichalcogenides. *Nat. Electron.* **4**, 495-501 (2021).
24 Li, N. *et al.* Large-scale flexible and transparent electronics based on monolayer molybdenum disulfide field-effect transistors. *Nat. Electron.* **3**, 711-717 (2020).
25 Pu, J. *et al.* Highly Flexible and High-Performance Complementary Inverters of Large-Area Transition Metal Dichalcogenide Monolayers. *Adv. Mater.* **28**, 4111-4119 (2016).
26 Tang, J. *et al.* Flexible CMOS integrated circuits based on carbon nanotubes with sub-10 ns stage delays. *Nat. Electron.* **1**, 191-196 (2018).
27 Chen, H. *et al.* Large-scale complementary macroelectronics using hybrid integration of carbon nanotubes and IGZO thin-film transistors. *Nat. Commun.* **5**, 4097 (2014).
28 Lei, T. *et al.* Low-voltage high-performance flexible digital and analog circuits based on ultrahigh-

purity semiconducting carbon nanotubes. *Nat. Commun.* **10**, 2161 (2019).
29 Sun, D. M. *et al.* Flexible high-performance carbon nanotube integrated circuits. *Nat. Nanotechnol.* **6**, 156-161 (2011).
30 Zhang, H. *et al.* High-Performance Carbon Nanotube Complementary Electronics and Integrated Sensor Systems on Ultrathin Plastic Foil. *ACS Nano* **12**, 2773-2779 (2018).
31 Xiang, L. *et al.* Low-power carbon nanotube-based integrated circuits that can be transferred to biological surfaces. *Nat. Electron.* **1**, 237-245 (2018).
32 Zhao, C. *et al.* Evaporated tellurium thin films for p-type field-effect transistors and circuits. *Nat. Nanotechnol.* **15**, 53-58 (2020).
33 Li, Y. V. *et al.* Low-Voltage Double-Gate ZnO Thin-Film Transistor Circuits. *IEEE Electron Device Letters* **34**, 891-893 (2013).
34 Jiang, C. *et al.* Printed subthreshold organic transistors operating at high gain and ultralow power. *Science* **363**, 719-723 (2019).
35 Lin, Z. *et al.* Solution-processable 2D semiconductors for high-performance large-area electronics. *Nature* **562**, 254-258 (2018).
36 Zhao, M. *et al.* Large-scale chemical assembly of atomically thin transistors and circuits. *Nat. Nanotechnol.* **11**, 954-959 (2016).
37 Dai, Z. *et al.* Large-Area Chemical Vapor Deposited MoS_2 with Transparent Conducting Oxide Contacts toward Fully Transparent 2D Electronics. *Adv. Funct. Mater.* **27**, 03119 (2017).
38 Wachter, S. *et al.* A microprocessor based on a two-dimensional semiconductor. *Nat. Commun.* **8**, 14948 (2017).
39 Xu, H. *et al.* High-Performance Wafer-Scale MoS_2 Transistors toward Practical Application. *Small* **14**, e1803465 (2018).
40 Tang, J. *et al.* In Situ Oxygen Doping of Monolayer MoS_2 for Novel Electronics. *Small* **16**, e2004276 (2020).
41 Wang, H. *et al.* Integrated circuits based on bilayer MoS_2 transistors. *Nano Lett.* **12**, 4674-4680 (2012).
42 Wang, H. *et al.* in *2012 International Electron Devices Meeting.* 4.6.1-4.6.4.
43 Huang, M. *et al.* Multifunctional high-performance van der Waals heterostructures. *Nat. Nanotechnol.* **12**, 1148-1154 (2017).
44 Li, Z. *et al.* High-performance heterogeneous complementary inverters based on n-channel MoS_2 and p-channel SWCNT transistors. *Nano Research* **10**, 276-283 (2017).
45 Li, W. *et al.* Uniform and ultrathin high-κ gate dielectrics for two-dimensional electronic devices. *Nat. Electron.* **2**, 563-571 (2019).
46 Pezeshki, A. *et al.* Static and Dynamic Performance of Complementary Inverters Based on Nanosheet alpha-$MoTe_2$ p-Channel and MoS_2 n-Channel Transistors. *ACS Nano* **10**, 1118-1125 (2016).
47 Zhang, Q. *et al.* Simultaneous synthesis and integration of two-dimensional electronic components. *Nat. Electron.* **2**, 164-170 (2019).
48 Kong, L. *et al.* Doping-free complementary WSe_2 circuit via van der Waals metal integration. *Nat. Commun.* **11**, 1866 (2020).
49 Tosun, M. *et al.* High-gain inverters based on WSe_2 complementary field-effect transistors. *ACS Nano* **8**, 4948-4953 (2014).
50 Chen, L. *et al.* Gigahertz Integrated Circuits Based on Complementary Black Phosphorus Transistors. *Adv. Electron. Mater.* **4**, 1800274 (2018).
51 Wu, P. *et al.* Two-dimensional transistors with reconfigurable polarities for secure circuits. *Nat. Electron.* **4**, 45-53 (2020).
52 Yu, L. *et al.* High-Performance WSe_2 Complementary Metal Oxide Semiconductor Technology and Integrated Circuits. *Nano Lett.* **15**, 4928-4934 (2015).
53 Lee, S. & Nathan, A. Subthreshold Schottky-barrier thin-film transistors with ultralow power and high intrinsic gain. Science 354, 302-304 (2016).
54 Zhao, Y. *et al.* Three-Dimensional Flexible Complementary Metal-Oxide-Semiconductor Logic Circuits Based On Two-Layer Stacks of Single-Walled Carbon Nanotube Networks. *ACS Nano* **10**, 2193-2202 (2016).
55 Ha, M. *et al.* Printed, sub-3V digital circuits on plastic from aqueous carbon nanotube inks. *ACS Nano* **4**, 4388-4395 (2010).
56 Long, G. *et al.* Carbon nanotube-based flexible high-speed circuits with sub-nanosecond stage delays. *Nat. Commun.* **13**, 6734 (2022).
57 Kim, Y.-H. *et al.* Flexible metal-oxide devices made by room-temperature photochemical activation

of sol–gel films. *Nature* **489**, 128-132 (2012).

58 Zhao, D. *et al.* Fast Flexible Plastic Substrate ZnO Circuits. *IEEE Electron Device Letters* **31**, 323-325, doi:10.1109/LED.2010.2041321 (2010).

59 Zschieschang, U. *et al.* Flexible low-voltage organic transistors and circuits based on a high-mobility organic semiconductor with good air stability. *Adv. Mater.* **22**, 982-985 (2010).

60 Borchert James, W. *et al.* Flexible low-voltage high-frequency organic thin-film transistors. *Sci. Adv.* **6**, eaaz5156 (2020).

61 Guo, Y. *et al.* Direct visualization of structural defects in 2D semiconductor. *Chin. Phys. B*, **31**, 076105 (2022).62 Li, Z. *et al.* Efficient strain modulation of 2D materials via polymer encapsulation. *Nat. Commun.* **11**, 1151, (2020).
