# Supplementary material for: Low power flexible monolayer MoS2 integrated circuits
Source: Nat Commun. 2023 Jun 19;14:3633. doi: 10.1038/s41467-023-39390-9 (PMC10279675; doi:10.1038/s41467-023-39390-9)
Supplement: Supplementary file 3 — Description of Additional Supplementary Files [file 41467_2023_39390_MOESM3_ESM.pdf]

Description of Additional Supplementary Files

File Name: Supplementary Movie 1

Description: Real time output signals of ring oscillator
